# Characterization and functional analysis of phytoene synthase gene family in tobacco

**DOI:** 10.1186/s12870-020-02816-3

**Published:** 2021-01-07

**Authors:** Zhaojun Wang, Lin Zhang, Chen Dong, Jinggong Guo, Lifeng Jin, Pan Wei, Feng Li, Xiaoquan Zhang, Ran Wang

**Affiliations:** 1 College of Tobacco Science, Henan Agricultural University, Zhengzhou, 450002 China; 2 China Tobacco Gene Research Center, Zhengzhou Tobacco Research Institute, Zhengzhou, 450001 China; 3 China Tobacco Yunnan Industrial Co., Ltd., Kunming, 650231 Yunnan China; 4 State Key Laboratory of Cotton Biology, Key Laboratory of Plant Stress Biology, School of Life Sciences, Henan University, 85 Minglun Street, Kaifeng, 475001 China; 5 School of Life Sciences, School of Agricultural Sciences, Zhengzhou University, No. 100 Science Road, Gaoxin Distract, Zhengzhou, 450001 Henan China

**Keywords:** Carotenoids, Phytoene synthase, Tobacco

## Abstract

**Background:**

Carotenoids play important roles in photosynthesis, hormone signaling, and secondary metabolism. Phytoene synthase (PSY) catalyzes the first step of the carotenoid biosynthetic pathway. In this study, we aimed to characterize the *PSY* genes in tobacco and analyze their function.

**Results:**

In this study, we identified three groups of *PSY* genes, namely *PSY1, PSY2*, and *PSY3,* in four *Nicotiana* species; phylogenetic analysis indicated that these genes shared a high similarity with those in tomato but not with those in monocots such as rice and maize. The expression levels of *PSY1* and *PSY2* were observed to be highest in leaves compared to other tissues, and they could be elevated by treatment with certain phytohormones and exposure to strong light. No *PSY3* expression was detected under these conditions. We constructed virus-induced *PSY1* and *PSY2* silencing in tobacco and found that the newly emerged leaves in these plants were characterized by severe bleaching and markedly decreased carotenoid and chlorophyll content. Thylakoid membrane protein complex levels in the gene-silenced plants were also less than those in the control plants. The chlorophyll fluorescence parameters such as Fv/Fm, ΦPSII, qP, and NPQ, which reflect photosynthetic system activities, of the gene-silenced plants were also significantly decreased. We further performed RNA-Seq and metabonomics analysis between gene-silenced tobacco and control plants. RNA-Seq results showed that abiotic stress, isoprenoid compounds, and amino acid catabolic processes were upregulated, whereas the biosynthesis of cell wall components was downregulated. Metabolic analysis results were consistent with the RNA-Seq. We also found the downstream genes in carotenoid biosynthesis pathways were upregulated, and putative transcription factors that regulate carotenoid biosynthesis were identified.

**Conclusions:**

Our results suggest that PSY can regulate carotenoid contents not only by controlling the first biosynthesis step but also by exerting effects on the expression of downstream genes, which would thereby affect photosynthetic activity. Meanwhile, PSY may affect other processes such as amino acid catabolism and cell wall organization. The information we report here may aid further research on *PSY* genes and carotenoid biosynthesis.

**Supplementary Information:**

The online version contains supplementary material available at 10.1186/s12870-020-02816-3.

## Background

Carotenoids are widely found in photosynthetic organisms, including plants, algae, and cyanobacteria. Chemically, carotenoids belong to isoprenoid compounds; typical carotenoids contain 40 carbon atoms (C40) that are formed by the condensation of eight C5 isoprenoid units. The number of conjugated double bounds in their chemical structure confers them a visible-light absorption property that produces their characteristic color of yellow to red [1, 2]. Carotenoids contain a large number of different components; at present, nearly 1200 natural carotenoids have been found in 700 organisms from all domains of life. Carotenoids that do not contain oxygen are classified as carotenes, and those that contain oxygen are classified as xanthophylls. In addition to the typical C40 carotenoids, some carotenoids that are shorter (C30) or longer (C45 or C50) have also been found [3].

Although humans do not metabolically synthesize carotenoids, carotenoids can be acquired via the consumption of food or supplementation. As naturally occurring pigments, carotenoids have a range of functions in human health. Carotenoids are important antioxidants as they absorb specific wavelengths of light and are the precursors of vitamin A. Moreover, they play important roles in protecting the eyes and in maintaining normal vision. Furthermore, they may protect against certain types of cancer by enhancing cell communication, suppressing abnormal cell growth, or providing UV protection. Carotenoids can prevent heart disease by reducing oxidized low-density lipoproteins [1, 4].

Carotenoids are also indispensable in plants. They provide protection against photooxidative damage; photoprotection is one of their most important functions. Under strong light conditions, carotenoids can dissipate excess energy as heat, eliminate free radicals, and prevent the lipid peroxidation of membranes, thereby enhancing the adaptation of plants to different light conditions [5]. Another important function of carotenoids is that it has a role in the reaction center of photosystem II. Carotenoids promote the formation of pigment-protein complexes and assist in energy absorption and electron flow transport [6]. In plants, carotenoids can serve as precursors to phytohormones such as abscisic acid (ABA) [7] and strigolactones [8], which both play vital roles in plant development and stress responses. Additionally, carotenoids play important roles in plant reproduction: the different colors that they give can attract animals that help in pollination and seed dispersal [9].

The biosynthesis of carotenoids in plants is part of the isoprenoid precursor metabolism. Starting from isopentenyl diphosphate (IPP) and dimethylallyl diphosphate (DMAPP), the biosynthesis of carotenoids is catalyzed by a series of enzymes [3]; the first step is the generation of geranylgeranyl diphosphate (GGPP) through the addition of three IPP molecules to one DMAPP, whose conversion is catalyzed by GGPP synthetase (GGPPS). GGPP is a precursor to several groups of other isoprenoids [10]. The next step in carotenoid biosynthesis is the production of 40-carbon phytoene through the condensation of two GGPP molecules; this condensation reaction is catalyzed by the enzyme phytoene synthase (PSY) and is considered the main “bottleneck” in the carotenoid biosynthetic pathway [11]. Then, phytoene is then converted to lycopene through a series of desaturation and isomerization reactions. Two types of phytoene desaturases, namely phytoene desaturase (PDS) [12] and ζ-carotene desaturase (ZDS) [13], are reportedly responsible for the desaturation reactions, whereas 15-cis-ζ-carotene isomerase (Z-ISO) catalyzes the isomerization reactions [14]. The next step is the cyclization of lycopene, wherein two branches, namely α- and β- branches, which both are converted into different components, are formed. The α-branch is relatively simple; lycopene is cyclized into δ-carotene with the help of lycopene ε-cyclase (LCYE) [15] and further cyclized into α-carotene by lycopene β-cyclase (LCYB) [16]. α-carotene can be hydroxylated by two types of carotenoid hydroxylases. Carotenoid β-hydroxylase (CHYB, mainly cytochrome P450 enzymes, CYP97 type) produce zeinoxanthin which is further hydroxylated by carotenoid ε-hydroxylase (CHYE, mainly CYP97C1) into lutein. Compared with the α-branch, the β-branch contains relatively more steps that lead to many intermediate products. First, lycopene undergoes two rounds of cyclization that is catalyzed by LCYB and leads to the production of γ- and β-carotene. Then, β-carotene undergoes two steps of hydroxylation reaction that is catalyzed by β-carotene hydroxylase (BCH) and leads to the production of β-cryptoxanthin and zeaxanthin [17]. Next, a two-step cyclization reaction of zeaxanthin is catalyzed by the enzyme zeaxanthin epoxidase (ZEP) and forms antheraxanthin and violaxanthin. The last step in the β-branch of the carotenoid biosynthetic pathway is the conversion of violaxanthin into neoxanthin; this conversion is catalyzed by neoxanthin synthase (NXS) [11].

PSY catalyzes the biosynthesis of phytoene from GGPP, which is a common precursor of many other isoprenoids [10]. The formation of phytoene is the first step in carotenoid biosynthesis and the main bottleneck step [18]. PSYs are encoded by small gene families; the genes encoding PSY have been identified and isolated in many species such as *Arabidopsis* [19], rice [20], maize [21], and tomato [22–24], and their function and expression patterns have been previously reported [25]. In *Arabidopsis*, the *PSY* gene is expressed in not only photosynthetic tissues but also non-photosynthetic tissues, including roots, in trace amounts and has a pattern of co-expression with other carotenoid pathway genes [26], indicating that the *PSY* gene is involved mainly in photosynthetic pathways. The expression of *PSY* genes is regulated by various factors, including developmental and environmental signals [27]. Phytohormones, especially ethylene, play an important role in the regulation of *PSY* gene expression; increased ethylene levels significantly upregulate the transcription of *PSY* genes [28]. Abscisic acid can also regulate the expression of *PSY* genes [25]. Environmental signals such as strong light, salt, drought, temperature, and photoperiod can also modify the expression levels of *PSY* genes [29]. Some important transcription factors were found to perceive the signals mentioned above and in turn control the transcription of *PSY* genes; for example, PHYTOCHROME INTERACTING FACTOR 1 (PIF1) [30] and LONG HYPOCOTYL 5 (HY5) [31], which belong to bHLH and bZIP families, respectively, were proven to be involved in the light-induced regulation of *PSY* gene expression. At the protein level, PSYs are also regulated; the regulation of PSYs include the localization of PSY within the chloroplast; this localization influence their bioavailability [32]. Furthermore, carotenoid metabolites have been found to negatively regulate PSY protein levels [33].

Similar to other plants, carotenoids also play an important role in photosynthesis, physiological processes, and stress responses in tobacco [34]. In addition, due to the properties of tobacco having huge biomass and being easy to genetically modified, tobacco is considered an ideal species from which to obtain valuable carotenoid components [35]. PSYs control the metabolic flux of carotenoids, making the functions of tobacco PSYs notable for studying. In a previous study, two transcripts were cloned from *Nicotiana tabacum* cultivar Petit Havana SR1 and showed 86% identity in both nucleotide and amino acid sequences [36]. The overexpression of both genes resulted in a severe dwarf phenotype, changes in pigment composition, and high levels of phytoene; these confirm the importance of the role of PSYs in controlling tobacco carotenoid biosynthesis. However, the two sequences were obtained by using homology-based cloning. The reference genome sequences of some *Nicotiana* species, such as *N. tabacum* [37, 38], *N. benthamiana* [39], *N. sylvestris*, and *N. tomentosiformis* [40] have been released. Thus, the aim of this study was to survey PSY coding genes at the genome level and extensively study their functions in carotenoid biosynthesis and photosynthesis such that more information about this gene family is obtained.

## Results

### Identification of *PSY* genes in tobacco

BLAST analysis was performed by querying *Arabidopsis* PSY protein sequences from different tobacco genomes, and 6, 5, 3, and 3 candidate *PSY* genes were found in *N. tabacum, N. benthamiana, N. sylvestris*, and *N. tomentosiformis,* respectively. Their temporary names and molecular characteristics are shown in Table 1. The coding sequence length of tobacco *PSYs* ranged from 924 to 1326 bp, and the resulting protein molecular weights ranged from 34.86 to 50.29 kD. The isoelectric point of PSYs ranged from 6.74 to 9.16 pH, indicating that these proteins are alkalescent. The exon number of *PSYs* ranged from six to eight (Table 1).

**Table 1:** *PSY* genes identified in four *Nicotiana* species

	Gene name	Gene ID	Exon number	MW (KDa)	PI	CDS (bp)	Length (aa)	Pfam Matches		
								ID	Start	End
*N. tabacum*	*NtPSY1–1*	mRNA_24760_cds	7	46.53	7.53	1233	410	PF00494	129	384
	*NtPSY1–2*	mRNA_28821_cds	7	46.56	8.1	1233	410	PF00494	129	384
	*NtPSY2–1*	mRNA_108630_cds	7	49.55	8.98	1323	440	PF00494	155	410
	*NtPSY2–2*	mRNA_3350_cds	7	49.72	9.16	1326	441	PF00494	156	411
	*NtPSY3–1*	mRNA_22099_cds	6	43.75	8.71	1146	381	PF00494	104	358
	*NtPSY3–2*	mRNA_111132_cds	6	43.83	8.51	1146	381	PF00494	103	358
*N. benthamiana*	*NibenPSY1–1*	Niben101Scf01959g00004	8	46.52	6.78	1233	410	PF00494	129	384
	*NibenPSY1–2*	Niben101Scf04020g00002	8	50.29	7.51	1326	441	PF00494	129	382
	*NibenPSY2*	Niben101Scf07253g01008	8	49.64	8.75	1323	440	PF00494	157	412
	*NibenPSY3–1*	Niben101Scf08679g04027	6	44.14	8.60	1146	381	PF00494	103	358
	*NibenPSY3–2*	Niben101Scf04118g01004	7	34.86	6.74	924	307	PF00494	83	252
*N. sylvestris*	*NsylPSY1*	mRNA_81209_cds	7	46.53	7.53	1233	410	PF00494	129	384
	*NsylPSY2*	mRNA_73510_cds	7	49.34	8.98	1317	438	PF00494	155	410
	*NsylPSY3*	mRNA_53352_cds	6	43.90	8.63	1146	381	PF00494	103	358
*N. tomentosiformis*	*NtomPSY1*	mRNA_60982_cds	7	46.56	8.10	1314	410	PF00494	129	384
	*NtomPSY2*	mRNA_59648_cds	7	49.51	9.16	1320	439	PF00494	156	411
	*NtomPSY3*	mRNA_83828_cds	6	43.83	8.51	1146	381	PF00494	103	358

The gene IDs shown were extracted from the genomic annotation information of each species deposited in Sol Genomics Network (SGN) database (https://solgenomics.net/). The genome version of *N. tabacum* used here was reported by Sierro et al., 2014 [37]

### Phylogenetic analysis of tobacco *PSY* gene family

**Fig. 1:**
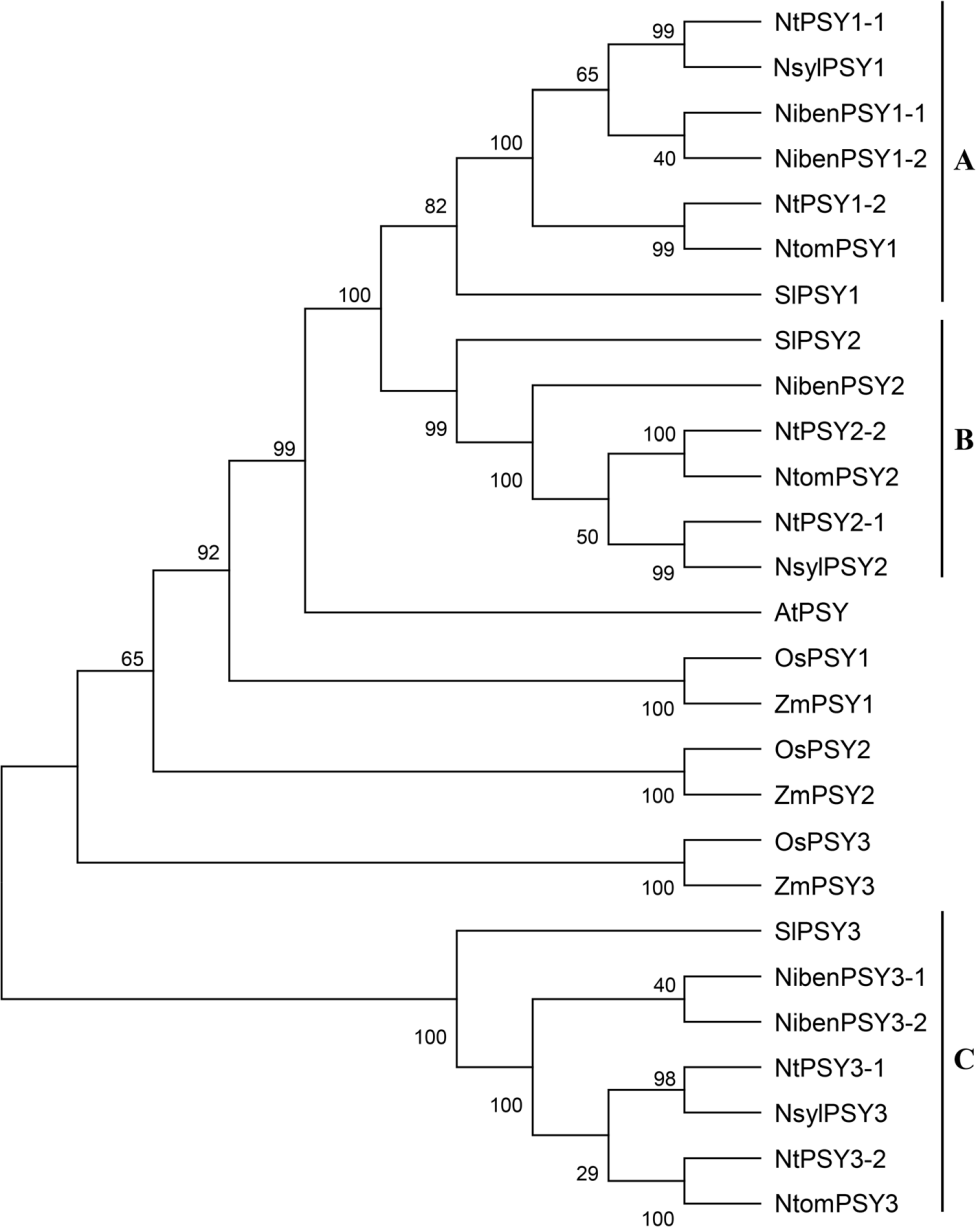
**Phylogenetic analysis of tobacco PSY protein sequences.** PSY protein sequences of *Arabidopsis*, rice, maize, and tomato using sequence accession numbers from a previous study [24] were downloaded from GeneBank database

The phylogenetic relationships of tobacco *PSY* genes and homologs in *Arabidopsis*, rice, maize, and tomato were analyzed using MEGA 5 software. *PSY* genes from different tobacco species can be divided into three groups (A, B, and C) based on their phylogenetic relationships (Fig. 1). Among them, *NtPSY1–1, NtPSY1–2, NibenPSY1–1, NibenPSY1–2, NsylPSY1*, and *NtomPSY1* were classified under group A, *NtPSY2–1, NtPSY2–2, NibenPSY2, NsylPSY2*, and *NtomPSY2* under group B, and *NtPSY3–1, NtPSY3–2, NibenPSY3–1, NibenPSY3–2, NsylPSY3*, and *NtomPSY3* under group C. In each group, strong correlations among the genes from *N. tabacum, N. sylvestris*, and *N. tomentosiformis*, compared with that of *N. benthamiana*, were observed. Under group A and B, 7 exons and 6 introns were identified in the *PSY* genes of *N. tabacum, N. sylvestris*, and *N. tomentosiformis*, whereas 8 exons and 7 introns were identified in the genes of *N. benthamiana*; for group C, 6 exons and 5 introns were identified in all the genes, except *NibenPSY3–2*, which contained 7 exons and 6 introns (Table 1). These results indicate a relatively low phylogenetic relationship between *N. benthamiana* and other *Nicotiana* species.

Three *PSY* genes in tomato were also clustered with tobacco *PSY* genes into three groups (Fig. 1), indicating that *PSY* gene sequences are conserved in Solanaceae species. However, *PSYs* in *Arabidopsis*, rice, and maize were not clustered with those in tobacco and tomato, suggesting that the sequences of *PSYs* among these species and Solanaceae were diverse.

### Cis-element analysis of *NtPSY* promoters

**Fig. 2:**
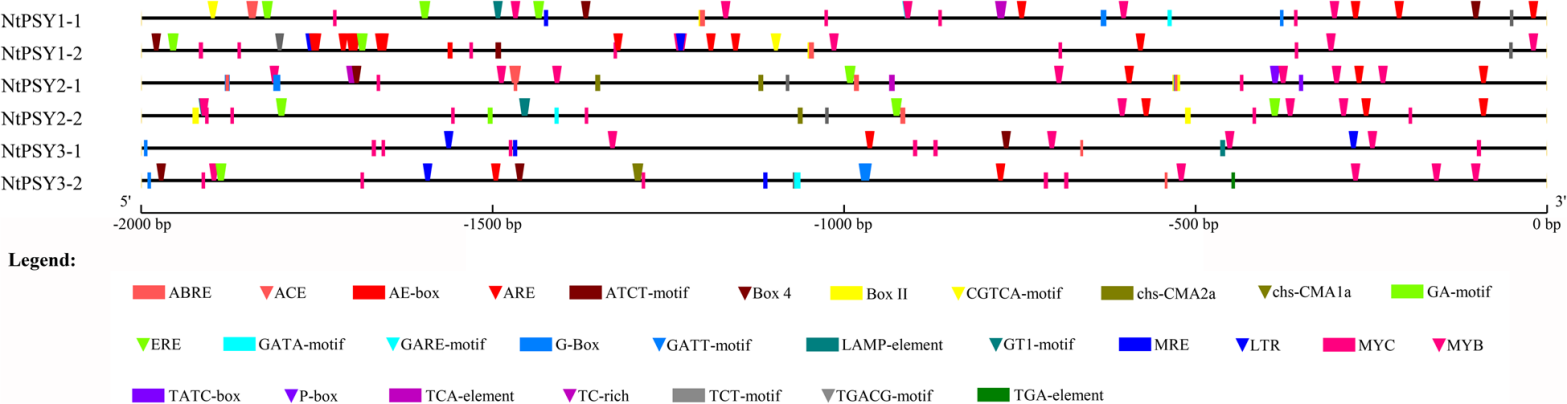
**Cis-elements of** ***NtPSY*** **gene promoter.** A fragment of 2000 bp upstream of the start codons of each *NtPSY* gene were analyzed. The core promoter elements such as TATA-box and CAAT-box were masked for clarity

We used *N. tabacum* as a model to survey cis-elements in tobacco *PSY* gene promoters. Fragments of 2000 bp upstream of the start codons of 6 *NtPSY* genes were extracted from tobacco genomic sequences and queried against PlantCARE database (http://bioinformatics.psb.ugent.be/webtools/plantcare/html/). As shown in Fig. 2, most of the cis-elements found were involved in responses to light (ACE, AE-box, ATCT-motif, Box 4, Box II, chs-CMA1a, chs-CMA2a, GA-motif, GATA-motif, GATT-motif, G-Box, LAMP-element, GT1-motif, MRE, and TCT-motif). Other cis-elements were identified to be involved in responses to temperature (LTR), drought stress (MYB, TC-rich), or phytohormones, including MeJA (CGTCA-motif, TGACG-motif), abscisic acid (ABRE), auxin (TGA-element), gibberellin (GARE-motif, TATC-box, P-box), and salicylic acid (TCA-element), indicating that the expression of *PSYs* is regulated by a wide range of developmental and environmental factors.

Among these cis-elements, box 4, GATA-motif, G-box, TCT-motif, MYC, ABRE, ERE, ARE, and MYB were present in all three groups of *NtPSY* (Additional file 1: Table S1). AE-box, ATCT-motif, TGACT-motif, CGTCA-motif, and GCN4-motif were present in only *NtPSY1*. chs-CMA2a, GA-motif, TATC-box, P-box, TCA-element, and W box were present exclusively in *NtPSY2*. chs-CMA1a, GATT-motif, LAMP-element, and TGA-element were solely present in *NtPSY3*. The diversity of cis-elements in different *NtPSY* promoters indicates that their expression may be regulated by different mechanisms.

### Expression pattern of *NtPSY* genes in tissues

**Fig. 3:**
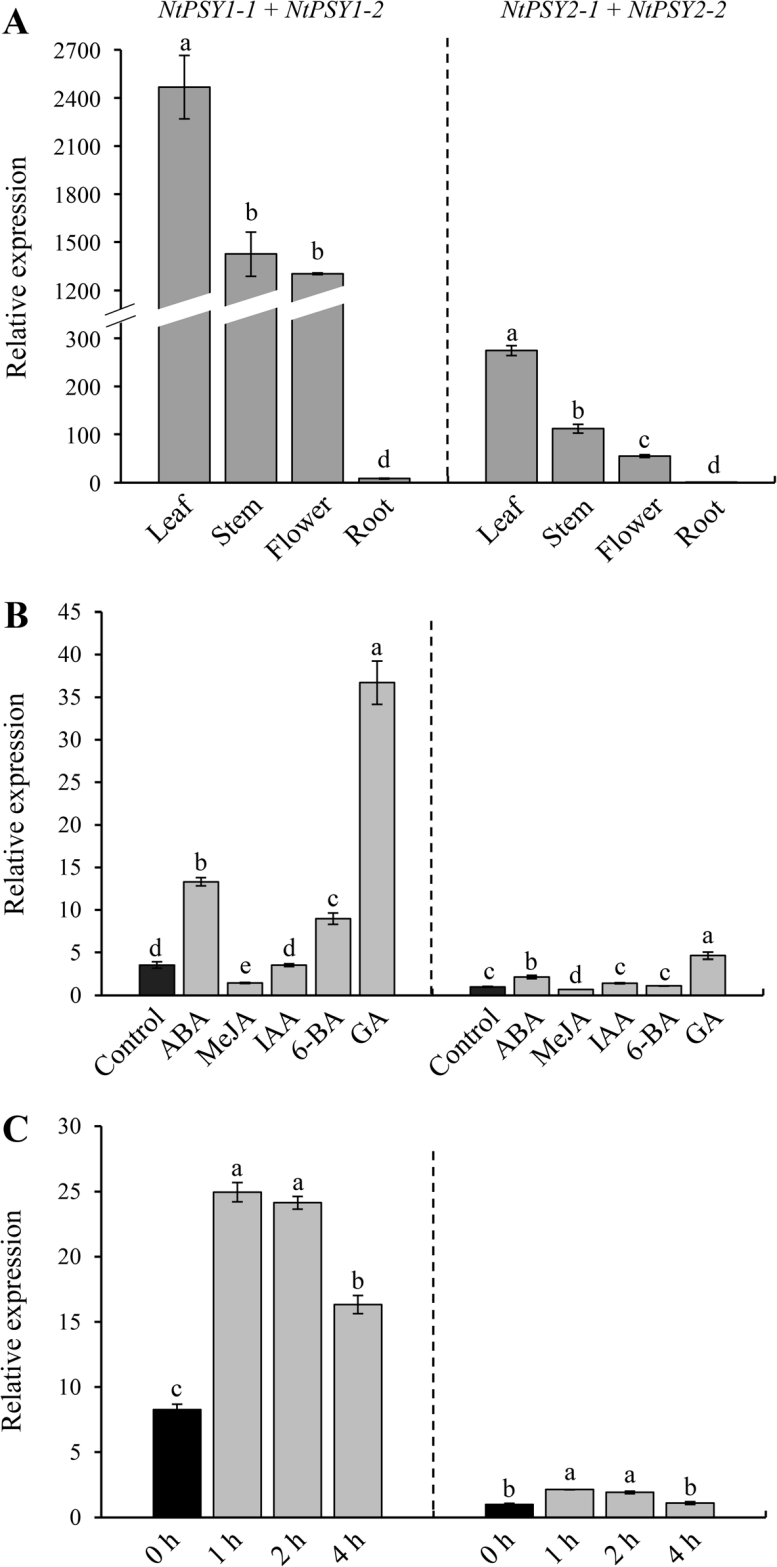
**Expression pattern of** ***NtPSY*** **genes. a** Expression levels of *NtPSY* genes in leaves, stems, flowers, and roots; the relative expression levels in each tissue were calculated by setting the expression value of *NtPSY2–1* + *NtPSY2–2* in roots as 1. **b** Changes in the expression of *NtPSY* genes under treatment with different phytohormones; the relative expression levels under different conditions were calculated by setting the expression value of *NtPSY2–1* + *NtPSY2–2* in the control as 1. **c** Changes in the expression of *NtPSY* genes under strong light conditions; the relative expression levels at different intervals were calculated by setting the expression value of *NtPSY2–1* + *NtPSY2–2* at 0 h as 1. Three pairs of conserved qPCR primers that can be used to estimate the sum expression of *NtPSY1–1* and *NtPSY1–2*, *NtPSY2–1* and *NtPSY2–2*, *NtPSY3–1* and *NtPSY3–2*, respectively, were designed. Columns and bars represent the means and standard errors (*n* = 3), respectively. Columns marked by different letters indicate statistical significance (*P* < 0.05). The data for the expression of *NtPSY3–1* and *NtPSY3–2* are not shown as no expression was detected under all the conditions

The gene expression levels of *NtPSY* in four tissues (leaf, stem, flower, and root) at full-bloom stage were compared. Due to the high similarity among *NtPSY* genes, three pairs of conserved qPCR primers (see Additional file 2: Table S2) that can be used to estimate the sum expression of *NtPSY1–1* and *NtPSY1–2*, *NtPSY2–1* and *NtPSY2–2*, and *NtPSY3–1* and *NtPSY3–2*, respectively, were designed. The results indicated that the expression of *NtPSY3–1* and *NtPSY3–2* was not detectable in any of the four tissues (data not shown), indicating that they possibly are not expressed in these tissues. Similar expression patterns were identified in the other four genes; the highest expression levels were found in leaves, intermediate levels in stems and flowers, and relatively low levels in roots (Fig. 3a), indicating that *NtPSY* genes function mainly in leaves, stems, and flowers. In addition, the expression levels of *NtPSY1–1* and *NtPSY1–2* were much higher than those of *NtPSY2–1* and *NtPSY2–2*, suggesting that *NtPSY1–1* and *NtPSY1–2* are functionally more important than *NtPSY2–1* and *NtPSY2–2*.

### The expression of *NtPSY* genes are influenced by different phytohormones and strong light conditions

To obtain the expression profiles of tobacco *PSY* genes under phytohormone treatment and strong light conditions, *N. tabacum* was used as a model, treated with abscisic acid (ABA), methyl jasmonate (MeJA), indole-3-acetic acid (IAA), 6-benzyladenine (6-BA), gibberellin (GA), and exposed to strong light. qPCR was performed to determine the relative expression levels of *NtPSY* genes under different treatments. No expression for *NtPSY3–1* and *NtPSY3–2* was detected after any of the treatments (data not shown). After the treatment of *N. tabacum* with ABA, 6-BA, and GA, the expression levels of the other four genes were significantly upregulated, but significantly downregulated in the *N. tabacum* treated with MeJA; no marked changes in expression levels were identified after IAA treatment (Fig. 3b). Under strong light conditions, the expression of *NtPSY1–1*, *NtPSY1–2*, *NtPSY2–1*, and *NtPSY2–2* were all upregulated and reached a peak after 1 h of treatment and declined thereafter (Fig. 3c). The expression levels of *NtPSY1–1* and *NtPSY1–2* were markedly higher than those of *NtPSY2–1* and *NtPSY2–2* under all treatments. These results indicate that phytohormones and light play important roles in regulating *NtPSY* gene expression*.*

### Virus-induced *NbibenPSY* gene silencing

**Fig. 4:**
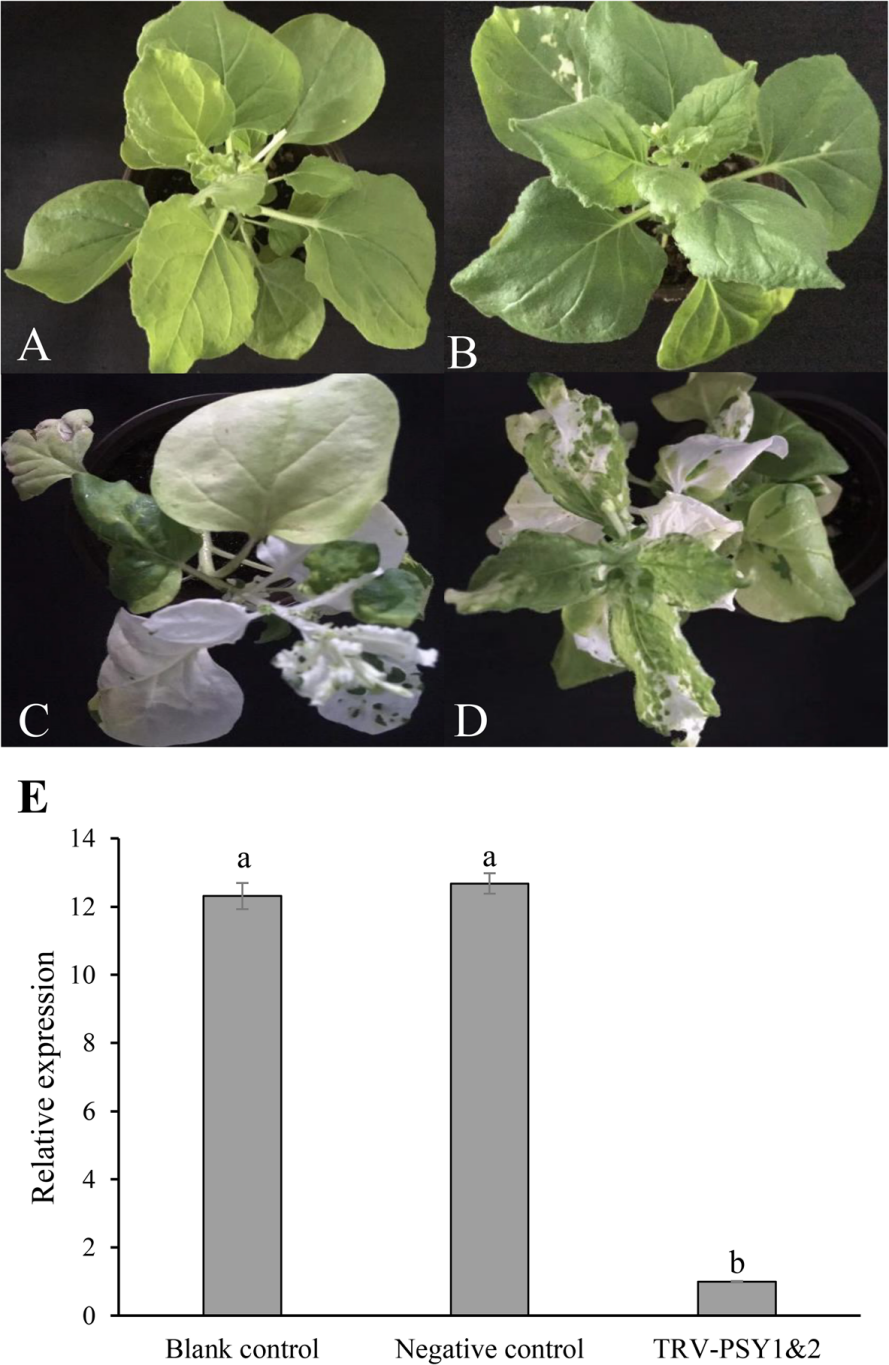
**TRV-mediated** ***PSY*** **gene silencing in** ***N. benthamiana*. a** Blank control (transformed using distilled water). **b** Negative control (transformed using empty vector). **c** Positive control (transformed using TRV-PDS construct). **d** TRV-PSY1&2 (co-transformed with TRV-PSY1 and TRV-PSY2). **e** Total expression of *NibenPSY1–1*, *NibenPSY1–2,* and *NibenPSY2* in blank control, negative control, and TRV-PSY1&2 measured by qPCR using conserved primers. The relative expression levels in different plants were calculated by setting the gene expression value in TRV-PSY1&2 as 1. Columns and bars represent the means and standard erorrs (*n* = 3), respectively. Columns marked by different letters indicate *P* < 0.05

To further investigate the function of tobacco *PSY* genes, we generated two virus-induced gene silencing constructs, namely TRV-PSY1 and TRV-PSY2. The former contains a conserved fragment shared by *NibenPSY1–1* and *NibenPSY1–2* and can silence both of them, whereas the latter can silence *NibenPSY2*. The two constructs were co-introduced into *N. benthamiana* by agrobacterium-mediated transformation to silence these three genes simultaneously; distilled water, empty vector, and TRV-PDS construct (can silence the phytoene desaturase gene as previously described [41]) were defined as blank, negative, and positive control, respectively. As shown in Fig. 4a-d, the newly emerged leaves of the positive control and TRV-PSY1/TRV-PSY2 co-transformed plants (named TRV-PSY1&2 hereafter) were severely bleached and characterized by abnormally wrinkled shapes, whereas no marked changes were identified in the blank and negative controls. Conserved qPCR primers were designed to estimate the expression levels of all three *NibenPSY* genes. The expression levels of *NibenPSY* genes was markedly suppressed in TRV-PSY1&2 plants compared to those of the blank and negative controls (Fig. 4e). These results suggest that the *NibenPSY* genes were silenced.

### Photosystem changes in *NbibenPSY*-silenced plants

**Fig. 5:**
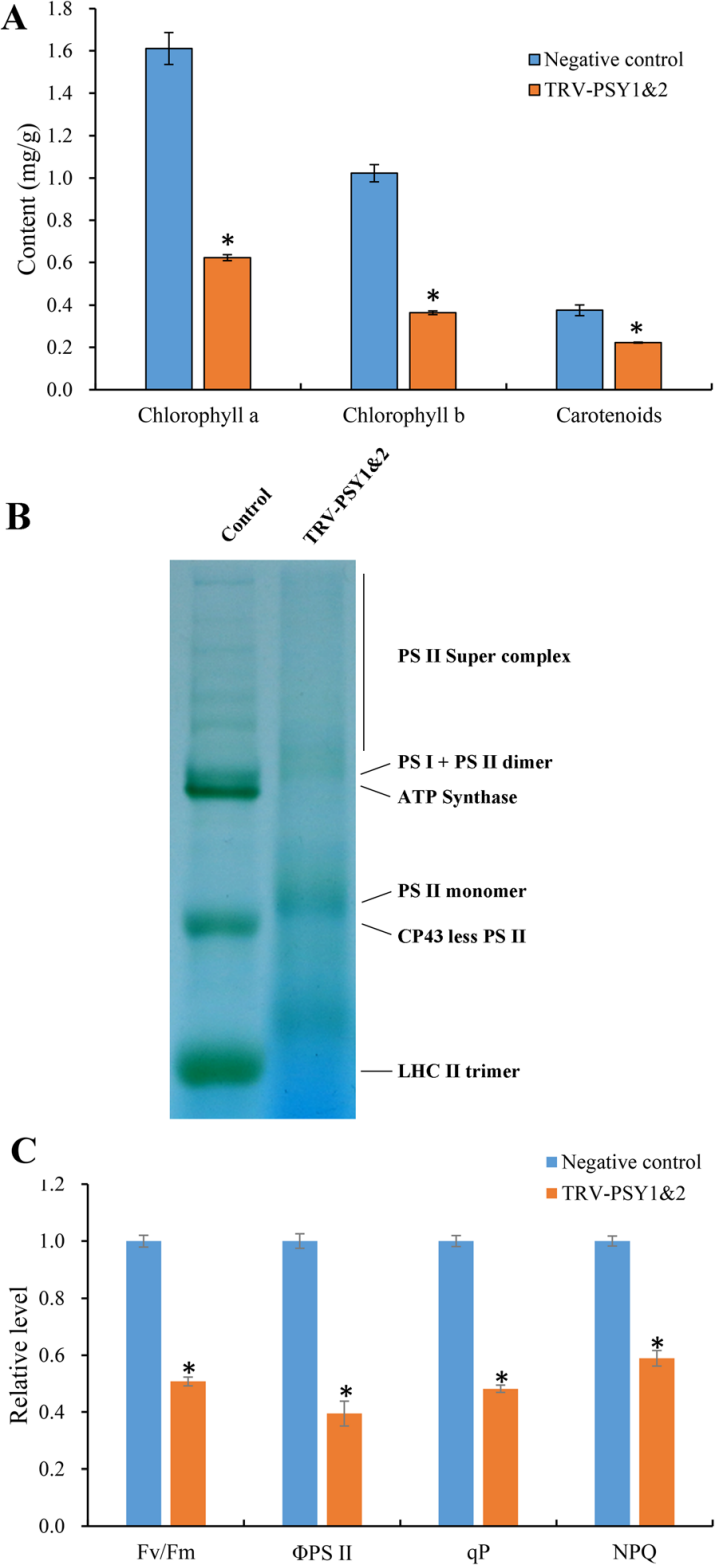
**Photosystem changes in virus-mediated** ***PSY*** **gene silencing in** ***N. benthamiana*. a** Carotenoid and chlorophyll content. **b** Blue-native polyacrylamide gel-electrophoresis analysis of thylakoid membrane protein complex in TRV-PSY1&2 and negative control plants. **c** Chlorophyll fluorescence difference between TRV-PSY1&2 and negative control plants; the relative levels of each parameter were calculated by setting the value in the negative control as 1. Columns and bars represent the means and standard errors (*n* = 9), respectively. * indicate *P* < 0.05. PS I, photosystem I. PS II, photosystem II. CP 43, 43 kD Chlorophyll a binding protein. LHC II, light harvesting complex II. Fv/Fm, maximum quantum efficiency of PSII photochemistry. ΦPSII, sum of the quantum yields of PSII photochemistry. qP, photochemical quenching. NPQ, nonphotochemical quenching

Carotenoids have been long proven to play important roles in plant photosynthesis [6], and the bleached phenotype of *NbibenPSY*-silenced plants we observed inspired us to analyze potential changes in photosystem. First, we measured carotenoid and chlorophyll content (Fig. 5a). Compared with the negative controls, the carotenoid content in TRV-PSY1&2 plants was significantly decreased, and only 60% carotenoids were detected. The contents of chlorophyll a and chlorophyll b were also decreased in TRV-PSY1&2 plants, with decrements of 67.26 and 64.65%, respectively.

Next, we explored the effects of *NbibenPSY* silencing on thylakoid structures; thylakoid membrane protein complex was analyzed using blue-native polyacrylamide gel-electrophoresis (BN-PAGE) (Fig. 5b). All protein band densities were decreased in TRV-PSY1&2 plants compared to the negative control, indicating that there is less accumulation of the thylakoid membrane protein complex in TRV-PSY1&2 plants.

We measured the chlorophyll fluorescence difference between TRV-PSY1&2 and negative control plants to evaluate their photosynthetic performance. Four parameters, namely Fv/Fm (maximum quantum efficiency of PSII photochemistry), ΦPSII (sum of the quantum yields of PSII photochemistry), qP (photochemical quenching), and NPQ (non-photochemical quenching), were measured (Fig. 5c); compared with the measurements of these parameters in the negative control, those in TRV-PSY1&2 plants were significantly decreased, indicating that the photosystem activities in *NbibenPSY*-silenced plants were significantly decreased.

### Metabolite analysis of TRV-PSY1&2 leaves compared with the control

The metabolic changes in leaves induced by the silencing of *PSY* genes were analyzed using GC-MS. The levels of 85 known metabolites were determined (Additional file 3: Table S3). Most of the compounds, including amino acids and organic acids, were downregulated in TRV-PSY1&2. Only 16 components were upregulated in TRV-PSY1&2; these components include cell wall components and mainly sugars and their derivatives, including arabinofuranose, levoglucosan, and arabinitol. Interestingly, sedoheptulose was also among the upregulated components. In plants, sedoheptulose exists mainly as monophosphate, plays vital roles during photosynthesis, and is liberated only upon cell death [42], indicating that cell death severely occurs in TRV-PSY1&2.

### Global analysis of RNA-seq data between TRV-PSY1&2 and control plants

To extensively analyze the function of tobacco PSYs, we performed an RNA-Seq analysis between the genes of TRV-PSY1&2 and negative controls. Three biological replicates were used for each group. Approximately 46 million paired-end raw reads were produced for each sample. Clean reads were obtained by discarding low-quality reads; a total of 270 million clean reads were generated and processed to assemble a de novo transcriptome using Trinity software [43]. A total of 418,816 transcripts were obtained, and each unigene was defined as the longest transcript in a homologous group. Finally, a total of 169,954 unigenes, with an average contig length of 598 bp and a minimum and maximum length of 201 and 12,283 bp, respectively, were obtained (Table 2).

**Table 2:** Length distribution of de novo assembled transcriptome contigs

	Total Nubmer	N50 (bp)	N90 (bp)	Maximum Length (bp)	Minimum Length (bp)	Average Length (bp)
Transcript	418,816	1269	340	12,283	201	820.65
Unigene	169,954	884	250	12,283	201	597.87

The unigenes obtained were queried against and annotated using the following databases: NT (NCBI nucleotide sequences), NR (NCBI non-redundant protein sequences), COG (Clusters of Orthologous Groups of proteins), KOG (euKaryotic Ortholog Groups), Swiss-Prot (A manually annotated and reviewed protein sequence database), TrEMBL, PFAM (Protein family), CDD (Conserved Domain Database), GO (Gene Ontology), and KEGG (Kyoto Encyclopedia of Genes and Genomes). All 169,954 unigenes were annotated; 64.14% of unigenes were annotated in at least one database and 0.82% in all databases (Additional file 4: Table S4).

The set of unigenes obtained above was used as a reference sequence, and clean reads of each sample were then mapped to it using Bowtie2 software [44]. For each sample, more than 93% of the clean reads were successfully mapped (Table 3), indicating that the quality of our results was sufficient for downstream analysis.

**Table 3:** Statistics of the RNA-Seq reads for TRV-PSY1&2 and control plants

	TRV_PSY_1	TRV_PSY_2	TRV_PSY_3	Control_1	Control _2	Control _3
Raw reads	42,416,708	59,297,192	39,934,284	56,175,286	40,652,118	38,640,458
Clean reads	41,528,460	58,008,450	39,041,364	53,804,542	39,789,524	37,816,924
Total mapped	37,172,244 (94.11%)	34,321,180 (94.01%)	48,743,371 (93.48%)	34,281,536 (94.28%)	36,413,818 (94.43%)	48,753,836 (93.36%)
Mutiple mapped	31,998,762 (81.01%)	29,594,117 (81.06%)	41,808,845 (80.18%)	29,636,940 (81.50%)	31,529,268 (81.76%)	41,082,440 (78.67%)
Uniquely mapped	5,173,482 (13.10%)	4,727,063 (12.95%)	6,934,526 (13.30%)	4,644,596 (12.77%)	4,884,550 (12.67%)	7,671,396 (14.69%)

TRV_PSY_1, TRV_PSY_2, and TRV_PSY_3 denote the three biological replicates for TRV-PSY1&2; Control_1, Control_2, and Control_3 are the three biological replicates for the negative control

To facilitate the comparison of differences in gene expression levels between different samples, the gene expression levels for each sample were calculated based on the reads mapping results and are shown as transcripts per million (TPM) values [45].

### Functional analysis of differentially expressed genes between TRV-PSY1&2 and control plants

**Fig. 6:**
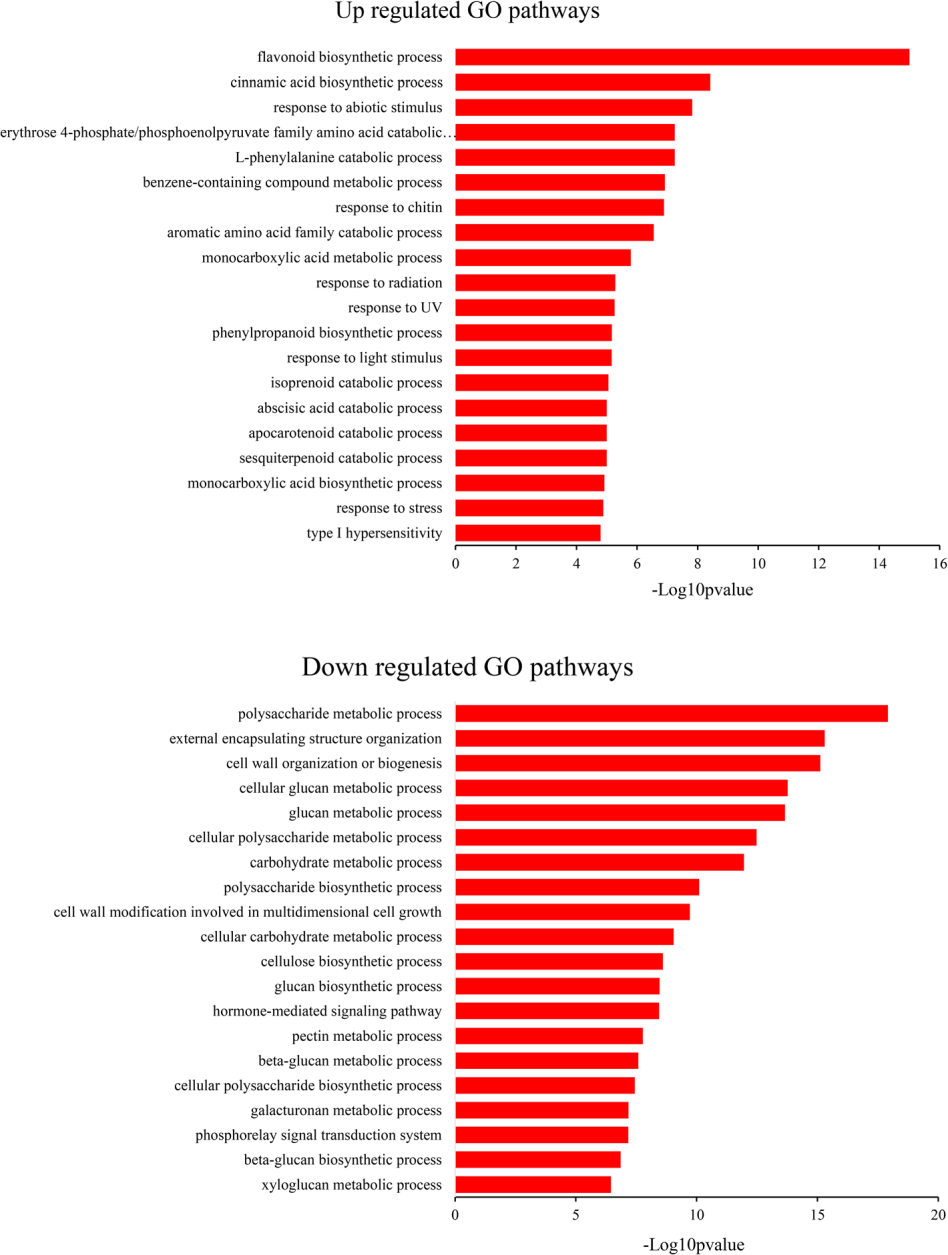
**Gene ontology enrichment analysis for differentially expressed genes between TRV-PSY1&2 and control plants.** Top 20 biological process gene ontology (GO) terms are shown here

Differentially expressed genes (DEGs) were identified using DESeq software [46], with *p-values* and *q-values* < 0.05 and log_2_FoldChange > 1 or < − 1 as the threshold for significant differential expression. In this study, a total of 748 and 854 DEGs were upregulated and downregulated in TRV-PSY1&2 plants, respectively (Additional file 5: Table S5). To evaluate the functional categories of these DEGs, GO enrichment analysis was performed using topGO software [47]. A *p* < 0.05 and *q* < 0.05 were set as the significant threshold, and 58 and 96 GO terms were enriched for these upregulated and downregulated DEGs, respectively (Additional file 6: Table S6). The top 20 biological process GO terms are shown in Fig. 6. The pathways involved in abiotic stress, isoprenoid compounds, and amino acid catabolic processes were upregulated in TRV-PSY1&2 plants, whereas the downregulated pathways were involved mainly in the biosynthesis of cell wall components, such as polysaccharides, glucans, cellulose, pectin, and galacturonan, indicating that PSY may play an important role in these processes.

### Changes in the expression of carotenoid biosynthesis pathway genes

The changes in the expression levels of *NbibenPSY* genes in gene-silenced and control plants were examined using the RNA-Seq data, which is consistent with the qPCR analysis (Fig. 4). Their expression was significantly repressed in TRV-PSY1&2 plants compared to that of the control plants (Table 4); this further confirms the high quality of the RNA-Seq results. GGPP synthetase, which operates upstream of PSY, was also downregulated in TRV-PSY1&2; on the contrary, almost all the downstream genes of PSY, except NXS, were upregulated in TRV-PSY1&2 compared to those of the control plants (Table 4).

**Table 4:** Expression levels of carotenoid biosynthesis pathway genes

	Gene ID	Expression level (TPM)	
		control	TRV-PSY1&2
GGPPS	TRINITY_DN36651_c0_g1	17.63	19.89
	TRINITY_DN35113_c0_g1	43.00	36.22
	TRINITY_DN69311_c0_g1	0.37	0.14
	TRINITY_DN37040_c0_g1	5.07	5.67
	TRINITY_DN40976_c1_g1	108.64	88.71
	TRINITY_DN39573_c2_g3	37.03	31.43
PSY	TRINITY_DN37717_c1_g3	19.94	6.48
	TRINITY_DN39386_c0_g1	106.77	60.14
	TRINITY_DN37717_c1_g2	62.61	26.49
PDS	TRINITY_DN43680_c0_g1	84.11	119.76
	TRINITY_DN43680_c0_g4	105.50	129.32
	TRINITY_DN43680_c0_g3	74.50	103.00
Z-ISO	TRINITY_DN36957_c0_g1	18.62	21.02
ZDS	TRINITY_DN38016_c0_g2	42.63	68.12
CRTISO	TRINITY_DN39955_c0_g1	19.45	21.73
β-LCY	TRINITY_DN38413_c1_g2	38.36	46.42
	TRINITY_DN33447_c0_g1	1.40	3.76
	TRINITY_DN33447_c0_g3	2.67	3.90
	TRINITY_DN33447_c0_g2	4.51	5.13
ε-LCY	TRINITY_DN44017_c2_g2	27.73	47.59
	TRINITY_DN44017_c2_g5	43.31	70.72
BCH	TRINITY_DN31514_c1_g2	1.33	1.42
	TRINITY_DN31514_c0_g1	6.51	18.88
	TRINITY_DN31514_c1_g1	1.66	1.41
CYP97A3	TRINITY_DN41762_c0_g1	72.38	91.75
	TRINITY_DN41762_c0_g5	36.55	51.21
	TRINITY_DN41762_c0_g2	9.60	12.30
CYP97B3	TRINITY_DN42530_c2_g4	13.25	18.58
	TRINITY_DN42530_c2_g2	10.87	15.26
CYP97C1	TRINITY_DN42272_c2_g1	25.72	30.82
	TRINITY_DN42272_c2_g6	43.38	41.64
ZEP	TRINITY_DN42020_c2_g1	105.06	108.99
	TRINITY_DN42020_c2_g2	2.36	2.50
NXS	TRINITY_DN32596_c0_g1	12.66	11.48
	TRINITY_DN36045_c5_g2	11.89	7.33
	TRINITY_DN35901_c3_g1	12.86	5.78
	TRINITY_DN36045_c5_g4	8.55	8.53
	TRINITY_DN39824_c2_g5	0.37	0.45

GGPPS, geranylgeranyl diphosphate synthase. PSY, phytoene synthase. PDS, phytoene desaturase. Z-ISO, ζ-isomerase. ZDS, ζ-carotene desaturase. CRTISO, carotenoid isomerase. β-LCY, lycopene β-cyclase. ε-LCY, lycopene ε-cyclase. BCH, carotenoid β-hydroxylase. CYP97A3, cytochrome P450 97A3. CYP97B3, cytochrome P450 97B3. CYP97C1, cytochrome P450 97C1. ZEP, zeaxanthin epoxidase. NXS, neoxanthin synthase. TPM, transcripts per million

### qPCR verification of carotenoid biosynthesis genes

To confirm the RNA-seq results, genes involved in the six steps in carotenoid biosynthesis pathways were randomly selected for qPCR analysis; the genes selected were those encoding GGPPS, PDS, ZDS, CRTISO, β-LCY, and NXS. For the determination of the total expression levels of genes in each of the selected steps, conserved primers were designed; the primers used are listed in Additional file 2: Table S2. The results indicate that although some quantitative differences at the expression level were present, qRT-PCR results indicated that all of the genes have similar expression patterns as indicated by the RNA-seq data (Additional file 7: Fig. S1), thereby further validating the RNA-Seq data.

### Identification of putative transcription factors that regulate carotenoid biosynthesis

As carotenoid biosynthesis pathway genes were elevated in TRV-PSY1&2 (Table 4), the implicated transcription factors among the DEGs may be involved in the regulation of carotenoid biosynthesis; in the upregulated and downregulated DEGs, 40 and 55 transcription factors, respectively, were identified (Additional file 8: Table S7). WRKY, MYB, and NAC were the top three upregulated transcription factor families, whereas ethylene-responsive transcription factor, bHLH, and WRKY were the top three downregulated transcription factor families. This indicates that they may induce the upregulation of carotenoid biosynthesis genes.

## Discussion

Carotenoids play important roles in photosynthesis, hormone signaling, and secondary metabolism. Phytoene synthase is known to play a significant role in the carotenoid biosynthetic pathway owing to its participation in the first committed step and the rate-limiting step, which potentially controls the downstream flux [18]. Even though only one *PSY* gene was found in *Arabidopsis* [19], many plant species are known to have multiple *PSY* genes with high sequence polymorphisms, including in rice [20], maize [21], and tomato [22–24], indicating a wide functional divergence in the *PSY* gene family of plant kingdom; thus, further information is still needed.

### *PSY* gene sequences are highly conserved among *Nicotiana* species and tomato

A previous study identified two *PSY* genes in *N. tabacum* using homology-based cloning [36]; however, in tomato, which is also of Solanaceae species, three *PSY* genes were found [24], suggesting that there may exist some other *PSY* genes in tobacco. In this study, we performed a whole genome screening to explore *PSY* genes in four *Nicotiana* species, namely *N. tabacum, N. benthamiana, N. sylvestris*, and *N. tomentosiformis*; 6, 5, 3, and 3 *PSY* genes (Table 1) were identified, respectively. Phylogenetic analysis showed that they can be divided into three groups (Fig. 1). Among them, *NtPSY1–1*, *NtPSY2–1*, and *NtPSY3–1* were highly correlated with *NsylPSY1*, *NsylPSY2*, and *NsylPSY3*, respectively. On the other hand, *NtPSY1–2*, *NtPSY2–2*, and *NtPSY3–2* were clustered more closely with *NtomPSY1*, *NtomPSY2*, and *NtomPSY3*, respectively. Considering that *N. tabacum* is an allotetraploid originating from the hybridization of *N. sylvestris* and *N. tomentosiformis* [37], we speculate that *NtPSY1–1*, *NtPSY2–1*, and *NtPSY3–1* are derived from *N. sylvestris*, whereas *NtPSY1–2*, *NtPSY2–2*, and *NtPSY3–2* originated from *N. tomentosiformis*. *N. benthamiana PSY* genes in each group showed relatively low similarity with those of the other three *Nicotiana* species, indicating a relatively lower phylogenetic relationship between *N. benthamiana* and other *Nicotiana* species. Notably, only one *PSY2* gene was found in *N. benthamiana*, which is also an allotetraploid [39]. The lack of the other *PSY2* member may be a result of gene loss during polyploidization.

The three *PSY* genes in tomato were also clustered into three groups (Fig. 1), indicating that tobacco *PSY* genes are homologs of those in tomato, and *PSY* gene sequences are conserved in Solanaceae species; however, *PSY* genes in *Arabidopsis*, rice, and maize were not clustered with those in tobacco and tomato, and this finding was also reported in a previous study [24], suggesting that the sequences of *PSY* genes are diverse among different species.

### *NtPSY1* has a dominant expression pattern relative to other *PSY* genes

In our results, tobacco *PSY1* and *PSY2* showed similar expression patterns, with the highest levels in leaves, intermediate in stems and flowers, and low in roots (Fig. 3a), suggesting that they function mainly in aerial tissues. The lack of difference in tissue-specific expression between tobacco *PSY1* and *PSY2* reduces the possibility of subfunctionalization between them. On the other hand, there may be functional redundancy between tobacco *PSY1* and *PSY2*. *PSY1* may have a dominant role in carotenoid biosynthesis, as its expression level is much higher than that of *PSY2*. This is quite different from tomato, as the three *PSY* genes work in different tissues, with *PSY1* mainly expressed in fruit [22], *PSY2* works in mature leaves [23], and *PSY3* functions in roots [24]. Many studies have found that PSY activity can be regulated at the post-transcriptional level [33, 48–50], which suggests that subfunctionalization between tobacco *PSY1* and *PSY2* may occur at the protein level; thus, the examination of protein location, catalytic activity, and relative protein content will provide more information about the function of different tobacco *PSY* genes.

On the other hand, the expression of *PSY3* was not detected in any of these tissues (data not shown), indicating that it does not work in these tissues. Tobacco *PSY3* belongs to a newly identified *PSY* clade, which is widespread but restricted to dicots [24]. Similar to our results, in *Manihot esculenta*, the *PSY3* transcripts were also absent in all the tissues and conditions tested [51]; however, in tomato, *PSY3* was strongly expressed exclusively upon root, mainly in response to phosphate starvation, whereas *Medicago truncatula PSY3* also works in roots, mainly involved in strigolactones biosynthesis and phosphate starvation [24]. Thus, a possible reason for the lack of expression of tobacco *PSY3* is that it is expressed only under special conditions, which is unknown now; however, other possible explanations also exist, for example, it may be a pseudo-gene. In summary, the functions of *PSY3* in dicots are far from being well known, and further studies are still needed.

The different expression patterns between tobacco *PSY* genes may be closely related to their promoter activity, which is supported by the different composition of cis-elements among different genes. As shown in Fig. 2 and Additional file 1: Table S1, some elements such as AE-box, ATCT-motif, TGACT-motif, CGTCA-motif*,* and GCN4-motif were present only in *NtPSY1*, which may be responsible for the high transcript levels of *NtPSY1*. Additionally, some cis-elements such as ACE, Box II, GT1-motif, and CAT-box were shared by *NtPSY1* and *NtPSY2*, but not in *NtPSY3*, which may explain the non-expression of *NtPSY3*. The cis-element that was solely present in the *NtPSY3* promoter was expected to support the cue for the regulation of *NtPSY3*. Four cis-elements, that is chs-CMA1a, GATT-motif, LAMP-element, and TGA-element were identified, which were involved in light and auxin response. However, no expression of *NtPSY3* was found under light and IAA treatment in our study, suggesting that they were non-functional.

Even though substantial differences were found between the expression levels of different *NtPSYs*, some cis-elements such as G-box and ABRE were found in all three groups of *NtPSY* (Additional file 1: Table S1). Some of these elements, such as G-box, play a vital role in light responsive expression of the *PSY* gene in *Arabidopsis* [52]. Thus, the different expression patterns of tobacco *PSY* genes should be regulated by only part of the elements identified in their promoters, and selective deletions of each cis-element are needed to demonstrate their detailed transcriptional regulation mechanism.

### Tobacco *PSY* genes play crucial role in photosynthesis and photoprotection by controlling the synthesis of carotenoids

Earlier studies have found that carotenoids are essential components of the photosynthetic system, reducing the carotenoid contents will dramatically decrease photosynthetic efficiency, leading to the albino phenotype [1]. The newly emerged leaves of TRV-PSY1&2 were also severely blenched (Fig. 4). Based on our results, we speculate that this phenotypic alteration mainly occurred at the metabolic level, but *NtPSY* was still the causal gene. The direct consequence of *NtPSY* silencing was the dramatic reduction in carotenoid content (Fig. 5a), which led to the instability of the light-harvesting complex (Fig. 5b) and reduced photosynthetic efficiency (Fig. 5c). In addition, the decline of the NPQ suggests that excess light energy could not be effectively dissipated, which exposed cells to severe oxidative stress [5], eventually leading to cell death and bleach of the leaves.

The consistent results of RNA-Seq and metabolic analysis further strengthen the important role of *PSY* genes in photosynthesis and photoprotection. Due to the reduction of photosynthetic efficiency, there will be insufficient energy for the cells; thus, many catabolic processes, including amino acids, isoprenoids, and sesquiterpenoids were upregulated in TRV-PSY1&2 (Fig. 6), consistent with this, metabonomics analysis showed that most of the metabolites were decreased in TRV-PSY1&2 (Additional file 3: Table S3). GO enrichment also found that pathways response to abiotic stimulus, like radiation, UV and light stimulus were up regulated, besides, flavonoid biosynthetic process was also up regulated, indicated that TRV-PSY1&2 is suffering severe stress caused by the excess light energy.

The down regulated GO pathways were mainly involved in the biosynthesis of cell wall components, like polysaccharide, glucan, cellulose, pectin and galacturonan (Fig. 6). Metabonomics analysis also showed that some cell wall components were increased in TRV-PSY1&2. More interestingly, the free sedoheptulose was also elevated, sedoheptulose was only liberated in dead plants [42], suggesting that much more cell death occurred in TRV-PSY1&2, which resulted in the disassembly of cell wall and increase of dissociative components. Similar to our results, it has also been found in tomato that knock down of *PSY-1* caused a wide reduction of housekeeping and structural proteins [53].

### Tobacco *PSY* genes are responsive to different phytohormones and light signal

Previous studies have found that the expression of *PSY* genes is regulated by various factors, for example, phytohormones such as ethylene and abscisic acid play important roles in the regulation of *PSY* gene expression. Environmental signals such as strong light, salt, drought, temperature, and photoperiod can also modify the expression level of PSY genes [27]. Transcription factors such as PIF1 and HY5 were found to perceive the signals mentioned above and in turn to control the transcription of *PSY* genes [31]. In this study, we also identified many cis-elements in *PSY* gene promoters, most of which were found to respond to the light signals, while phytohormone responsive elements were also found (Fig. 2). Consistent with this, we tested the effects of phytohormones and strong light stress on *PSY* expression, and found that ABA, 6-BA, and GA treatment could increase the expression of *PSY1* and *PSY2* (Fig. 3b). The strong light stress could also elevate *PSY1* and *PSY2* expression levels (Fig. 3c), indicating that similar to other plant species, tobacco *PSY1* and *PSY2* were regulated by these factors. To our surprise, most cis-elements found in *PSY1* and *PSY2* promoters were also present in the *PSY3* promoter, but *PSY3* showed no response to these treatments we tested, suggesting that *PSY3* may work in some other unknown processes.

### Tobacco *PSYs* work synergistically with other genes to control the carotenoids biosynthesis

As the first enzyme of the carotenoid biosynthesis pathway, *PSY* has been co-expressed with many photosynthesis-related genes, such as the biosynthesis of carotenoids and chlorophylls [26], which could explain the decrease in chlorophyll content in TRV-PSY1&2 plants (Fig. 5). Furthermore, in our RNA-Seq analysis, we also found that carotenoid biosynthesis genes were coordinated expressed in tobacco, as shown in Table 4. Most of the downstream genes in the carotenoid biosynthesis pathway were upregulated in TRV-PSY1&2 plants, suggesting that *PSY* could influence the expression of these genes. Consistent with our results, in tomato transgenic lines that overexpressed *PSY-1*, most of the downstream genes were suppressed at the transcriptional level [49]. Contrary to the changes in downstream genes, *GGPPS*, which works up stream of *PSY* and is responsible for the precursors of carotenoid biosynthesis, was downregulated in TRV-PSY1&2 plants (Table 4). Similar to our results, overexpression of tomato *PSY-1* elevated the transcript level of *GGPPS* [49]. Another study found that enhanced PSY activity could upregulate DXS levels [18]. DXS is an MEP pathway enzyme that also works up stream of PSY and response for the biosynthesis of isoprenoids, indicating that changes in *PSY* level could also influence the expression of the upstream genes. In tomato, PSY could be associated with other enzymes such as GGPPS into large protein complexes [48], suggesting that this association may influence the co-regulation of these genes.

Previous studies have identified a common ATCTA-motif in the promoter of some carotenoid biosynthesis genes*,* including *PSY* and *PDS*, and their upstream genes *DXS* and *HDR* in the MEP pathway. This motif is a binding site of ERF transcription factor [52, 54]. In this study, we identified 95 transcription factors among the DEGs (Additional file 8: Table S7). Among them, 15 belonged to the ERF family, indicating that they may be involved in the regulation of the coordinated expression between carotenoid biosynthesis genes, which needs further verification.

## Conclusions

We identified three groups of *PSY* genes in four *Nicotiana* species, which shared high similarity with those in tomato, but not with those in monocots. *PSY1* and *PSY2* showed the highest expression levels in leaves, and could be elevated by phytohormones and strong light treatment, but no expression of *PSY3* was detected. The photosynthetic system activity were significantly decreased in *PSY1* and *PSY2* silencing plants. RNA-Seq analysis showed that tobacco *PSYs* work synergistically with other genes to control carotenoid biosynthesis. The information obtained here may aid further research on *PSY* genes and carotenoid biosynthesis.

## Methods

### Plant materials and growth conditions

*Nicotiana benthamiana* and common tobacco (*Nicotiana tabacum* L.) variety K326 were used in this study. Seeds were germinated on moist soil and grown under 16 h light, 8 h dark, and 25 °C conditions.

### Identification of *PSY* genes in tobacco genomes

The genome sequences and annotation information of K326, *Nicotiana benthamiana, Nicotiana sylvestris*, and *Nicotiana tomentosiformis* were obtained from Sol Genomics Network (SGN) database (https://solgenomics.net/). The *Arabidopsis* PSY protein sequence (At5g17230) was obtained from the The *Arabidopsis* Information Resource (TAIR) database (https://www.arabidopsis.org/) and used as a query sequence to screen PSY sequences in various tobacco species using BlastP program and e-value < 1e^− 10^ as the query threshold. A PSY domain (accession PF00494) was extracted from Pfam database (http://pfam.xfam.org/) to determine PSY sequences using HMMER web server (https://www.ebi.ac.uk/Tools/hmmer/) [55].

### Phylogenetic analysis

To elucidate the phylogenetic relationship between tobacco PSY proteins and those of other species, phylogenetic analysis was conducted using MEGA 5 software [56]. The sequences and corresponding sequence accession numbers of PSY proteins in *Arabidopsis*, rice, maize, and tomato were used as previously described [24] and downloaded from GeneBank database (https://www.ncbi.nlm.nih.gov/).

Multiple sequence alignments of amino acid sequences were performed using the CLUSTALW algorithm using default parameters, and the resulting aligned region was used for phylogenetic analysis by Neighbor-Joining method [57], and the phylogenetic tree was constructed with 1000 bootstrap replicates.

### Cis-element analysis of tobacco *NtPSY* gene promoters

The 2000 bp sequence upstream of the start codons of *NtPSY* genes was obtained from the SGN database, and cis-element analysis was performed using PlantCARE web tools (http://bioinformatics.psb.ugent.be/webtools/plantcare/html/). The results obtained were visualized using of GSDS2.0 web server (http://gsds.cbi.pku.edu.cn/).

### Treatment with phytohormones and exposure to strong light

At the fifth-leaf stage, K326 plants were separately sprayed with 50 μmol/L gibberellic acid (GA), 100 μmol/L methyl jasmonate (MeJA), 10 μmol/L abscisic acid (ABA), 2 μmol/L 6-benzyladenine (6-BA), or 5 μmol/L 3-indoleacetic acid (IAA), the control plants were sprayed with double distilled water; leaves were harvested 8 h after treatment. Strong light conditions was defined as 1200 μmol∙m^− 2^∙s^− 1^ and the control conditions as 400 μmol∙m^− 2^∙s^− 1^, and samples were harvested at 0 h and, 1, 2, and 4 h after treatment. Three biological replicates were used for each treatment. The harvested materials were immediately submerged under liquid nitrogen and stored at − 80 °C until use.

### Virus-induced *NbibenPSY* silencing

To elucidate the biological functions of tobacco PSY, *NbibenPSY* genes were silenced using virus-induced gene silencing methods. A 684-bp cDNA fragment, which showed 96.2% similarity with *NbibenPSY1–1* and *NbibenPSY1–2*, was selected for simultaneous gene silencing. Another cDNA fragment of 726 bp was selected for *NbibenPSY2* silencing. The two fragments were obtained by PCR amplification using a template of leaf cDNA. The primers used are listed in Additional file 2: Table S2, with the restriction sites of *Kpn* I and *Xho* I as the cloning sites for forward and reverse primers, respectively.

The two fragments and the empty pTRV2 (pYL156) vector (described in Liu et al., [58]) were digested separately using *Kpn* I and *Xho* I restriction enzymes. Then, the fragments were ligated into digested pYL156 vectors, and confirmed by sequencing. Thus two constructs were obtained, namely TRV-PSY1 and TRV-PSY2. The two constructs were then transferred into *Agrobacterium tumefaciens* strain GV3101 using freeze-thaw method.

The infiltration of *N. benthamiana* leaves was performed mainly based on previously described methods [59]. Briefly, *A. tumefaciens* strains containing TRV-PSY1 or TRV-PSY2 were grown at 28 °C in Luria Bertani (LB) medium containing appropriate antibiotics. The cells were harvested and resuspended in the infiltration buffer (10 mm MES, pH = 5.5, 200 μm acetosyringone, and 10 mM MgCl_2_) to a final absorbance (optical density (OD) at 600 nm) of 1.0 and incubated for 2 h at 25 ± 2 °C. For leaf infiltration, each *A. tumefaciens* strain containing TRV-PSY1 or TRV-PSY2 were mixed in a 1:1 ratio in infiltration buffer and infiltrated into lower leaves using a 1 ml needleless syringe. The empty pYL156 vector and its derivative, TRV-PDS construct (could silence the *phytoene desaturase* gene as described previously [41]) were used as negative and positive controls, respectively, using the same method. The infiltrated plants were maintained at 25 °C for effective viral infection and spread.

### Photosynthetic activity measurement

The isolation of carotenoid and chlorophyll was performed as previously described [60]. Briefly, 50 mg (fresh weight) samples were mixed and shook with 1 ml 80% (v/v) ice-cold acetone in the dark at 4 °C for 30 min. After centrifugation (10,000×g, 2 min, 4 °C), absorbances at 663 nm, 647 nm, and 470 nm were recorded using a spectrophotometer, and pigment levels were calculated using the following equation: chlorophyll a = 12.25*A663–2.79*A647; chlorophyll b = 21.50*A647–5.10*A663; chlorophyll total = 7.15*A663 + 18.71*A647; carotenoids = (1000*A470–1.82* chlorophyll a – 85.02* chlorophyll b)/198.

Chlorophyll fluorescence was measured using Dual-PAM 100 (WALZ, Germany); four parameters, namely Fv/Fm (maximum quantum efficiency of PSII photochemistry), ΦPSII (sum of the quantum yields of PSII photochemistry), qP (photochemical quenching), and NPQ (non-photochemical quenching) were measured.

For thylakoid isolation, 1 g samples was put in 5 ml extracting buffer (500 mM sorbitol, 50 mM Tris-HCl, 2 mM EDTA, 1 mM MgCl_2_, and 1 mM MnCl_2_, pH = 6.8, 4 °C) ground, and filtered through a cell filter. Then, the mixture was centrifuged at 4 °C, 8000 g for 5 min. The thylakoids in the supernatant were then washed using 25BTH20G buffer (pH = 7.0, 20% glycerol, and 25 mM Bis-Tris), and centrifuged at 4 °C, 15,000×g for 5 min. Blue-native polyacrylamide gel electrophoresis (BN-PAGE) was performed as previously described [61] with some modifications; 12 μg chlorophyll was incubated with 1% β-DM, and the solubilized fraction was then loaded on a native gradient gel (5–12% (w/v), acrylamide/bisacrylamide ratio 32:1) topped with a 4% (w/v) stacking gel (ratio 1:4). After electrophoresis, the native gel was treated for 1.5 h with Laemmli buffer (138 mM Tris–HCl [pH 6.8], 6 M urea, 22.2% [v/v] glycerol, 4.3% [w/v] SDS, and 200 mM DTT), and the separated protein complexes were transferred onto a polyvinylidene fluoride membrane using Turbo Transfer system (Bio-Rad).

### Leaf metabolomics analysis

The metabolic profile of tobacco leaves from control and TRV-PSY1&2 was investigated using gas chromatography-mass spectrometry (GC-MS) according to previously described methods [62] with some modifications. The freeze-dried tissue was ground to a uniform powder and filtered using a 40-mesh sieve. Leaf powder (10 mg) was added to a 2 ml Eppendorf tube and soaked in 1.5 ml extraction solvent containing isopropanol/acetonitrile/water (3/3/2, v/v/v) with 25 μl (0.1 mg/ml) tridecanoic acid as an internal standard. All extracts were sonicated for 1 h and centrifuged for 10 min (14,000 rpm, 4 °C). Four-hundred μl the supernatant was transferred to a new tube and dried under nitrogen flow on an N-EVAP nitrogen evaporator. To increase the volatility of the metabolites, silylation reaction was performed by adding 100 μl methyl-trimethyl-silyl-trifluoroacetamide (MSTFA) to the sample and incubating it for 60 min at 60 °C.

GC-MS analysis of the metabolomic analysis was performed on Agilent 7683B series injector (Agilent, Santa Clara, CA) coupled to an Agilent 6890 N series gas chromatography system and 5975 mass selective detector (MSD) (Agilent, Santa Clara, CA). Agilent DB-5MS column (0.25 μm, 0.25 mm × 30 m, Agilent Technologies, Inc., Santa Clara, CA) was used. The column temperature was set at 70 °C for the first 4 min and then increased at 5 °C/min to 310 °C for 15 min. Helium (99.9995%) was used as the carrier gas. The column flow was 1.2 ml/min, and the column was equipped with a linear velocity control model. The temperatures of the interface and the ion source were adjusted to 280 °C and 230 °C, respectively. The electron impact (EI) model was set to achieve ionization of the metabolites at 70 eV.

Student’s *t*-test was used to determine the significant differences between the metabolites in the control and TRV-PSY1&2 (SPSS software, version 17.0).

### RNA preparation

Different plant tissues were harvested and immediately frozen using liquid nitrogen. Total RNA was isolated using Spin Column Plant Total RNA Purification Kit (Sangon Biotech, China). The quality and quantity of these RNA samples were further determined using Nanodrop 2000 (Thermo Fisher, US) and agarose gel electrophoresis.

### Reverse transcription and quantitative real-time PCR (qPCR) analysis

First-strand cDNA was synthesized using PrimeScript reverse transcriptase (Takara). The primers used for qPCR are listed in Additional file 2: Table S2. qPCR was performed using Roche Light-Cycler 480 System. The reaction mixture contained 2 μl primers (2.5 μM), 10 μl SYBR Green I Master Mix (Roche), 2 μl cDNA template, and 6 μl water. The real-time PCR conditions were set as follows: 95 °C for 5 min, followed by 45 cycles of 95 °C for 10 s, 60 °C for 30 s, and 72 °C for 20 s. A melting curve was established by slow heating from 60 °C to 95 °C throughout 20 min. Relative gene expression levels were calculated using 2^-ΔΔCT^ with three replicates for each sample. Data are presented as means ± standard deviation (SD) (*n* = 3).

### RNA-seq and data processing

Six samples were used for the RNA-seq analyses: three from the TRV-PSY1 and TRV-PSY2 co-infiltrate plants (TRV-PSY1&2) and three from the control plants. Total RNA was sent to Sangon Biotech (Shanghai) Co., Ltd., where the libraries were produced. The cDNA libraries were then sequenced using the Illumina HiSeq™ 2000. Then, 150-bp paired-end clean data were obtained by excluding the adaptors and low-quality reads using Trimmomatic software [63]. The clean reads generated were processed using Trinity software [43] to assemble a de novo transcriptome and used as a reference sequence for downstream analysis.

Unigenes obtained from the de novo transcriptome were queried against and annotated using the following databases: NT (NCBI nucleotide sequences), NR (NCBI non-redundant protein sequences), COG (Clusters of Orthologous Groups of proteins), KOG (euKaryotic Ortholog Groups), Swiss-Prot (A manually annotated and reviewed protein sequence database), TrEMBL, PFAM (Protein family), CDD (Conserved Domain Database), GO (Gene Ontology), and KEGG (Kyoto Encyclopedia of Genes and Genomes).

The set of unigenes obtained above was used as a reference sequence, and clean reads of each sample were then mapped to the sequence using Bowtie2 software [44]. Gene expression levels were calculated based on the reads mapping results and shown as transcripts per million (TPM) value [45].

We used the DESeq software [46] to identify differentially expressed genes (DEGs) between samples. An adjusted *p*-value < 0.05 found by DESeq were applied as standards to characterize the significance of gene expression levels. To identify the pathways significantly affected by the *PSY* genes, GO enrichment pathway analysis of DEGs was performed using topGO software [47].

## Supplementary Information

**Additional file 1: Table S1**.docx Cis-regulatory elements found in the promoter region of *NtPSY* genes.

**Additional file 2: Table S2**.docx Primer sequences used in qPCR analysis. The underlined letters indicate the manually added cloning site adaptors: *Kpn I* and *Xho* I for forward and reverse primers, respectively.

**Additional file 3: Table S3**.xlsx Relative metabolite levels in negative control and TRV-PSY1&2 leaves.

**Additional file 4: Table S4**.docx Summary of unigene annotation.

**Additional file 5: Table S5**.xlsx Differentially expressed genes between TRV-PSY1&2 and control plants

**Additional file 6: Table S6**.xlsx Gene ontology enrichment of differentially expressed genes between TRV-PSY1&2 and control plants

**Additional file 7: Figure S1**.tif qRT-PCR confirmation of carotenoid biosynthesis genes in TRV-PSY1&2 and control plants. GGPPS, geranylgeranyl diphosphate synthase. PDS, phytoene desaturase. ZDS, ζ-carotene desaturase. CRTISO, carotenoid isomerase. B-LCY, lycopene β-cyclase. NXS, neoxanthin synthase. Columns and bars represent the means and standard errors (*n* = 3), respectively. * indicates *P* < 0.05.

**Additional file 8: Table S7**.xlsx Differentially expressed transcription factors between TRV-PSY1&2 and control plants

## Data Availability

The RNA-seq datasets used this article are available in the NCBI Sequence Read Archive (SRA) (https://www.ncbi.nlm.nih.gov/sra/) under BioProject accession: PRJNA631583. The data that support the results are included within the article and its additional files.

## Abbreviations

ABA: Abscisic acid
BN-PAGE: Blue-native polyacrylamide gel-electrophoresis
DEG: Differentially Expressed Gene
GA: Gibberellin
GC-MS: Gas chromatography-mass spectrometry
GGPP: Geranylgeranyl diphosphate
IAA: Indole-3-acetic acid
MeJA: Methyl Jasmonate
PSY: Phytoene synthase
6-BA: 6-benzyladenine
