# Supplementary material for: Characterization and functional analysis of phytoene synthase gene family in tobacco
Source: BMC Plant Biol. 2021 Jan 7;21:32. doi: 10.1186/s12870-020-02816-3 (PMC7791662; doi:10.1186/s12870-020-02816-3)
Supplement: Supplementary file 1 — Additional file 1: Table S1.docx Cis-regulatory elements found in the promoter region of NtPSY genes. [file 12870_2020_2816_MOESM1_ESM.docx]

Table S1. Cis-regulatory elements found in the promoter region of *NtPSY* genes.

| Motif | Function | *NtPSY1* | *NtPSY2* | *NtPSY3* |
| --- | --- | --- | --- | --- |
| ACE | Light responsive element | + | + | - |
| AE-box |  | + | - | - |
| ATCT-motif |  | + | - | - |
| Box4 |  | + | + | + |
| Box II |  | + | + | - |
| chs-CMA1a |  | - | - | + |
| chs-CMA2a |  | - | + | - |
| GA-motif |  | - | + | - |
| GATA-motif |  | + | + | + |
| G-box |  | + | + | + |
| GATT-motif |  | - | - | + |
| LAMP-element |  | - | - | + |
| GT1-motif |  | + | + | - |
| MRE |  | + | - | + |
| TCT-motif |  | + | + | + |
| TGACG-motif | MeJA-responsiveness | + | - | - |
| CGTCA-motif |  | + | - | - |
| MYC |  | + | + | + |
| TGA-element | Auxin-responsive element | - | - | + |
| ABRE | Abscisic acid responsiveness | + | + | + |
| ERE | Ethylene-responsive element | + | + | + |
| GARE-motif | Gibberellin-responsive element | + | - | - |
| TATC-box |  | - | + | - |
| P-box |  | - | + | - |
| TCA-element | Salicylic acid responsiveness | - | + | - |
| ARE | Anaerobic induction response | + | + | + |
| LTR | Low-temperature responsiveness | + | - | + |
| W box | Abiotic stress responsiveness | - | + | - |
| MBS | MYB binding site involved in drought-inducibility | - | + | + |
| TC-rich | Defense and stress responsiveness | + | + | - |
| MYB | MYB protein binding site | + | + | + |
| GCN4_motif | Involved in endosperm expression | + | - | - |
| CAT-box | Involved in meristem expression | + | + | - |

+: Present, -: Absent.
