# Supplementary material for: Characterization and functional analysis of phytoene synthase gene family in tobacco
Source: BMC Plant Biol. 2021 Jan 7;21:32. doi: 10.1186/s12870-020-02816-3 (PMC7791662; doi:10.1186/s12870-020-02816-3)
Supplement: Supplementary file 2 — Additional file 2: Table S2.docx Primer sequences used in qPCR analysis. The underlined letters indicate the manually added cloning site adaptors: Kpn I and Xho I for forward and reverse primers, respectively. [file 12870_2020_2816_MOESM2_ESM.docx]

**Table S2 Primer sequences used in qPCR analysis**

| Primer | Sequence ( 5' - 3' ) | Usage |
| --- | --- | --- |
| TRV-PSY1-F | GCGGTACCTACACCGGCGGGAGAAATGGC | Contruction of TRV2-PSY1 vector |
| TRV-PSY1-R | GCCTCGAGCTTCTGGCATCTTCTCCGAC |  |
| TRV-PSY2-F | GCGGTACCAACGCATCACACATAACTCC | Contruction of TRV2-PSY2 vector |
| TRV-PSY2-R | GCCTCGAGAGAGGAAGTTCTATTAGGGG |  |
| qPCR-PSY1-F | GAAGCCGGAGATCCCTCTCC | qPCR analysis of *PSY1-1* and *PSY1-2* in *N. tabacum* and *N. benthamiana* |
| qPCR-PSY1-R | TTGCCCAAATAGCCCTTCTTC |  |
| qPCR-PSY2-F | GGCAGCTGAGATCTACCGATG | qPCR analysis of *PSY2-1* and *PSY2-2* in *N. tabacum* and *N. benthamiana* |
| qPCR-PSY2-R | TGCACATACTTCGCCACAACG |  |
| qPCR-PSY3-F | AGATGAACTTGTCGATGGACC | qPCR analysis of *PSY3-1* and *PSY3-2* in *N. tabacum* and *N. benthamiana* |
| qPCR-PSY3-R | GCATATCGGCTTTTCCTCG |  |
| qPCR-PSY-1&2-F | GATGATTTAGAAGTGAAGCCGG | qPCR analysis of *NibenPSY1-1, NibenPSY1-2* and *NibenPSY2* |
| qPCR-PSY-1&2-R | GCACCACACATATATTGCCCA |  |
| qPCR-GGPPS-F | TGGCATTTTTGGCTACCATTTCT | qPCR analysis of *GGPPS* gene |
| qPCR-GGPPS-R | GGCCTGGATTTTCTTGGGGA |  |
| qPCR-PDS-F | ATAAACCCTGACGAGCTTTC | qPCR analysis of *PDS* gene |
| qPCR-PDS-R | AATATGTTCAACAATCGGCAT |  |
| qPCR-CRTISO-F | CGTGTACACCGAGAATATGATG | qPCR analysis of *CRTISO* gene |
| qPCR-CRTISO-R | GTAGGCGAGAGTCAAGCACTC |  |
| qPCR-ZDS-F | TGAAATAGGGGAGCTTGATTTCCGC | qPCR analysis of *ZDS* gene |
| qPCR-ZDS-R | GAGCATATGCGACAGGATCCCAC |  |
| qPCR-β-LCY-F | GACAATACAACTAAAGATCTTGATAG | qPCR analysis of *β-LCY* gene |
| qPCR-β-LCY-R | CATAAGCTACTTGATATCCAGGAT |  |
| qPCR-NXS-F | GCCGGGCTCTATTCGACGTGAT | qPCR analysis of *NXS* gene |
| qPCR-NXS-R | ACTCTACCATATGGTCTTCCCAAAT |  |
| 26S-F | GAAGAAGGTCCCAAGGGTTC | qPCR analysis of *26S rRNA* gene (internal control) |
| 26S-R | TCTCCCTTTAACACCAACGG |  |

The underlined letters indicated the manually added cloning site adaptors, *Kpn I* and *Xho* I for forward and reverse primers, respectively.
