# Supplementary material for: Characterization and functional analysis of phytoene synthase gene family in tobacco
Source: BMC Plant Biol. 2021 Jan 7;21:32. doi: 10.1186/s12870-020-02816-3 (PMC7791662; doi:10.1186/s12870-020-02816-3)
Supplement: Supplementary file 4 — Additional file 4: Table S4.docx Summary of unigene annotation. [file 12870_2020_2816_MOESM4_ESM.docx]

**Table S4 Summary of unigene annotation**

| Database | Number of genes | Percentage (%) |
| --- | --- | --- |
| Annotated in CDD | 34786 | 20.47 |
| Annotated in KOG | 24524 | 14.43 |
| Annotated in NR | 65124 | 38.32 |
| Annotated in NT | 98594 | 58.01 |
| Annotated in PFAM | 24062 | 14.16 |
| Annotated in Swissprot | 46334 | 27.26 |
| Annotated in TrEMBL | 59765 | 35.17 |
| Annotated in GO | 53995 | 31.77 |
| Annotated in KEGG | 2492 | 1.47 |
| Annotated in at least one database | 109001 | 64.14 |
| Annotated in all database | 1402 | 0.82 |
| Total genes | 169954 | 100 |
