# Supplementary figures and images for: Characterization and functional analysis of phytoene synthase gene family in tobacco
Source: BMC Plant Biol. 2021 Jan 7;21:32. doi: 10.1186/s12870-020-02816-3 (PMC7791662; doi:10.1186/s12870-020-02816-3)

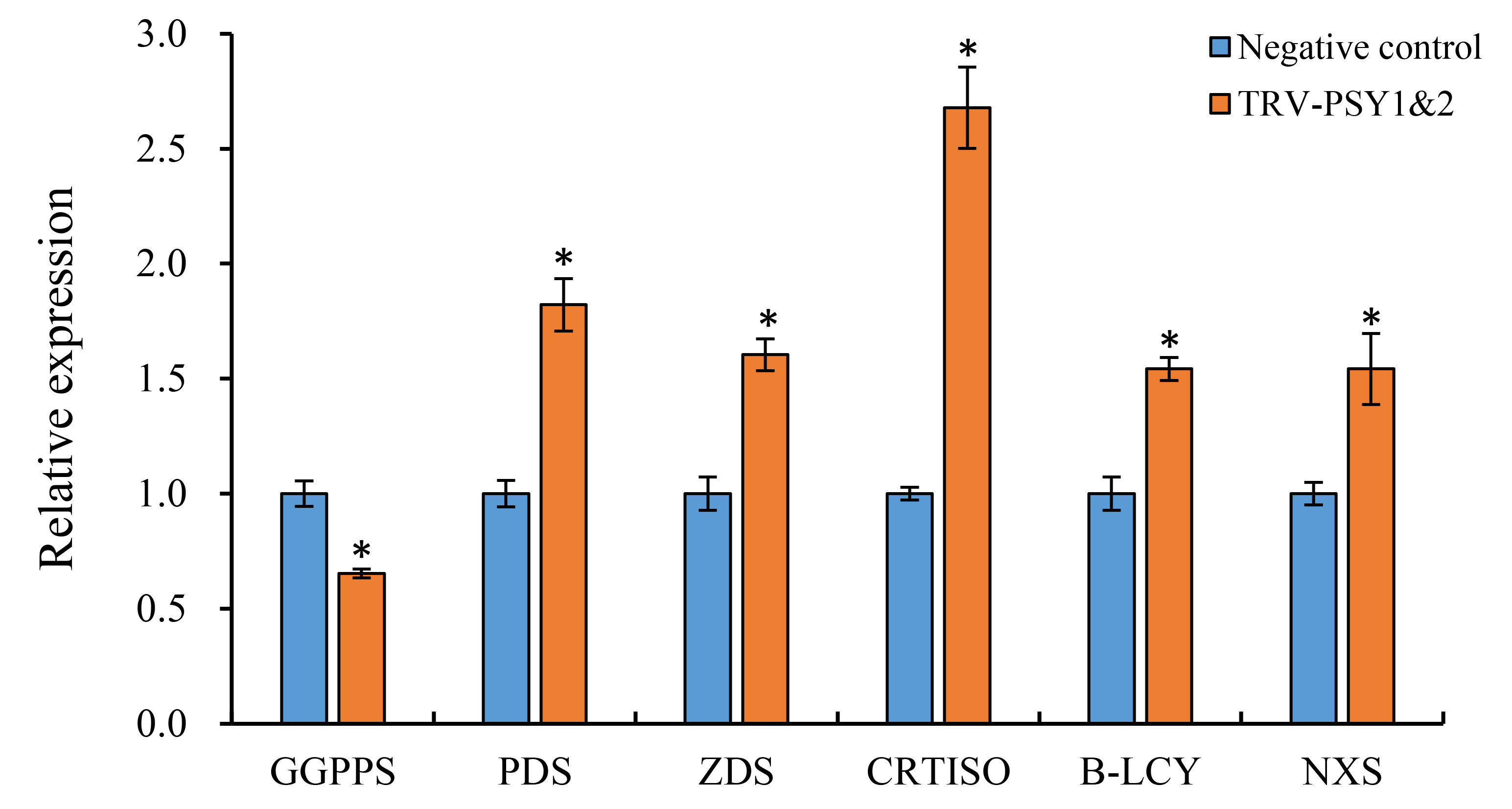

Supplement: Supplementary file 7 — Additional file 7: Figure S1.tif qRT-PCR confirmation of carotenoid biosynthesis genes in TRV-PSY1&2 and control plants. GGPPS, geranylgeranyl diphosphate synthase. PDS, phytoene desaturase. ZDS, ζ-carotene desaturase. CRTISO, carotenoid isomerase. B-LCY, lycopene β-cyclase. NXS, neoxanthin synthase. Columns and bars represent the means and standard errors (n = 3), respectively. * indicates P < 0.05. [file 12870_2020_2816_MOESM7_ESM.tif]
